# Bone Marrow Sparing by Intensity Modulated Proton Beam Therapy in Postoperative Irradiation of Gynecologic Malignancies

**DOI:** 10.1177/15330338241252622

**Published:** 2024-06-06

**Authors:** Antje Wark, Anil Gupta, Eva Meixner, Laila König, Juliane Hörner-Rieber, Tobias Forster, Kristin Lang, Malte Ellerbrock, Klaus Herfarth, Jürgen Debus, Nathalie Arians

**Affiliations:** 1 214788 Department of Radiation Oncology, Heidelberg University Hospital, Heidelberg, Germany; 2 Heidelberg Institute of Radiation Oncology (HIRO), Heidelberg, Germany; 3 National Center for Tumor Diseases (NCT), Heidelberg, Germany; 4 28730 All India Institute of Medical Sciences (AIIMS), New Delhi, India; 5 Heidelberg Ion-Beam Therapy Center (HIT), 27178 Heidelberg University Hospital, Heidelberg, Germany; 6 Clinical Cooperation Unit Radiation Oncology, German Cancer Research Center (DKFZ), Heidelberg, Germany; 7 German Cancer Consortium (DKTK), Heidelberg, Germany

**Keywords:** uterine cervical cancer, cervical cancer, uterine cancer, hematotoxicity, chemoradiotherapy, volumetric modulated, radiatio dosimetry, toxicity, proton therapy, insufficiency fracture

## Abstract

**Purpose:** The aim of this matched-pair cohort study was to evaluate the potential of intensity-modulated proton therapy (IMPT) for sparring of the pelvic bone marrow and thus reduction of hematotoxicity compared to intensity-modulated photon radiotherapy (IMRT) in the setting of postoperative irradiation of gynaecological malignancies. Secondary endpoint was the assessment of predictive parameters for the occurrence of sacral insufficiency fractures (SIF) when applying IMPT. **Materials and Methods:** Two cohorts were analyzed consisting of 25 patients each. Patients were treated with IMPT compared with IMRT and had uterine cervical (n = 8) or endometrial cancer (n = 17). Dose prescription, patient age, and diagnosis were matched. Dosimetric parameters delivered to the whole pelvic skeleton and subsites (ilium, lumbosacral, sacral, and lower pelvis) and hematological toxicity were evaluated. MRI follow-up for evaluation of SIF was only available for the IMPT group. **Results:** In the IMPT group, integral dose to the pelvic skeleton was significantly lower (23.4GyRBE vs 34.3Gy; *p* < 0.001), the average V_5Gy_, V_10Gy_, and V_20Gy_ were reduced by 40%, 41%, and 28%, respectively, compared to the IMRT group (*p* < 0.001). In particular, for subsites ilium and lower pelvis, the low dose volume was significantly lower. Hematotoxicity was significantly more common in the IMRT group (80% vs 32%; *p *= 0009), especially hematotoxicity ≥ CTCAE II (36% vs 8%; *p *= 0.037). No patient in the IMPT group experienced hematotoxicity > CTCAE II. In the IMPT cohort, 32% of patients experienced SIF. Overall SIF occurred more frequently with a total dose of 50.4 GyRBE (37.5%) compared to 45 GyRBE (22%). No significant predictive dose parameters regarding SIF could be detected aside from a trend regarding V50Gy to the lumbosacral subsite. **Conclusion:** Low-dose exposure to the pelvic skeleton and thus hematotoxicity can be significantly reduced by using IMPT compared to a matched photon cohort. Sacral insufficiency fracture rates appear similar to reported rates for IMRT in the literature.

## Introduction

Bone marrow (BM) is a vital organ responsible for the production of blood cells, playing a critical role in maintaining the body's overall hematopoietic function. It is a dynamic tissue that houses hematopoietic stem cells capable of self-renewal and differentiation into various blood cell lineages. More than one-half of the body's BM is located in the lumbar spine and pelvic skeleton.^1^ There is a direct correlation between radiation dose of bone and depletion of marrow,^2–5^ which can be easily measured by granulocytopenia, thrombocytopenia, and anemia. The vulnerability of BM to radiation poses a significant challenge, especially in the treatment of malignancies located in the pelvis.

Radiation therapy remains an indispensable part of treatment in gynecological malignancies such as uterine cervical cancer and endometrial cancer. Postoperative irradiation plays a crucial role in reducing the risk of locoregional recurrence and improving overall survival rates in these malignancies if risk factors are present.^6–9^ However, conventional photon-based radiation therapy techniques also affect the surrounding healthy tissues including the pelvic BM which can result in acute hematologic toxicities and can cause immune suppression.^10^ Radiation-induced myelosuppression can also lead to delayed or missed chemotherapy especially limiting the ability to deliver concurrent chemotherapy which might impact overall survival.^8^ Irradiation treatment of the pelvis can further lead to changes in bone mineral density as well as pelvic insufficiency fractures, most commonly affecting the sacral bone.^11^

Models of normal tissue complications have validated the idea that hematologic toxicity increases with the amount of pelvic BM exposed to radiation.^5,12^ Newer techniques in photon radiotherapy such as intensity-modulated radiotherapy (IMRT) including volumetric arc therapy (VMAT) have introduced the possibility of decreasing doses to the pelvic skeleton.^13–15^ However, the dose reduction is primarily seen in high dose (>35 Gy) volume by the cost of increase in low dose (0-30 Gy) volume and integral dose. Since low dose volume to the BM is associated with acute hematological toxicities,^16,17^ a satisfactory reduction of hematological toxicity could not be reached by these techniques.

Recent advancements in radiation therapy, such as proton beam therapy, have shown promising results in minimizing radiation-induced toxicities by precise delivery of radiation to the area of interest while sparing surrounding critical organs. Intensity-modulated proton beam radiotherapy (IMPT) is a special technique of proton radiotherapy akin to IMRT, which allows single or multifield optimization by using an inverse treatment planning system guided by a magnetically active raster scanning proton radiotherapy technique. IMPT has shown promising results in sparing gastrointestinal and genitourinary organs without compromising target volume coverage.^18,19^ The approach of preservation of BM function by BM sparing through IMPT has the potential to reduce treatment interruptions especially with concurrent chemotherapy, improve patients’ quality of life, and reduce long-term treatment-related complications for gynecological cancer patients.

This study aims to explore the potential of IMPT in regard to BM sparing during postoperative irradiation of gynecologic malignancies and to evaluate any dosimetric advantages compared to IMRT in reducing the doses to the pelvic skeleton and thus potentially hematotoxicity.

Ethics approval for the study was granted by the Heidelberg University ethics committee on December 11th, 2019 (#S-808/2019).

## Material and Methods

A total of 50 patients with cervical or endometrial cancer treated with postoperative radiotherapy at the Heidelberg Ion Therapy Center and the Department of Radiation Oncology of Heidelberg University Hospital between 2017 and 2023 were retrospectively selected for this comparative study.

The experimental cohort of this pair-matched cohort study consisted of 25 patients treated by IMPT within the APROVE trial.^20^ The APROVE trial was a prospective phase-II trial evaluating the safety and treatment tolerability of postoperative radiation using proton beam therapy with scanning technique. Primary endpoint of the APROVE trial was the lack of any acute GI or GU toxicity ≥ grade 3. All patients received postoperative radiotherapy (45-50.4 GyRBE) for endometrial (n = 17) or cervical (n = 8) carcinoma.

In order to select a control cohort of 25 patients treated by volumetric arc therapy (VMAT) with corresponding dose and diagnostic characteristics all patients treated by VMAT between 2017 and 2023 were screened. The selection of the pairs was done very carefully with exact matching regarding cancer type, prescription dose and simultaneous chemotherapy. Age was taken into account, although only approximately matched. Patients who received simultaneous integrated boost irradiation of lymph nodes were excluded. In each cohort, one patient received irradiation of the paraaortal region. Sample size was limited by the number of patients treated by IMPT.

Radiation therapy planning was performed with computed tomography (CT) scans in supine position using an immobilization device (ProSTEP by IT V, Innsbruck, Austria) for consistent patient positioning. Target volume definition, dose constraints to organs at risk and plan optimization were performed in accordance with the RTOG consensus guidelines^21^ while adhering to the ALARA (as low as reasonably achievable) principle. No BM-sparing objectives were implemented, neither for treatment by VMAT nor IMPT.

IMPT planning was performed using Syngo RT Planning System (Siemens, Erlangen, Germany), which applies a pencil beam algorithm for dose calculation. To account for geometrical uncertainties and physical beam inaccuracies a planning target volume (PTV) was created according to clinic internal standards by applying a margin of 5 mm isotropically and 7 mm in beam direction. Treatment planning was carried out by inverse planning employing two posterior oblique fields (160° and 200°) using an active raster-scanning technique. A third field (180°) was used in 13 cases (52%) to achieve coverage of at least 95% of the PTV. To increase dose robustness mainly single-beam optimization (SBO) was performed. Further details on delineation, constraints, planning, and treatment delivery technique can be found in a previous publication.^22^ Detailed description of the treatment facility can be found elsewhere.^23^ The reported doses for proton radiotherapy were scaled using a relative biological effectiveness (RBE) factor of 1.1.

VMAT plans were calculated as dual-arc VMAT using RayStation® 11B (Stockholm, Sweden) using Monte Carlo algorithm and delivered with 6MV linear accelerators. Radiotherapy planning and follow-up were done as per institutional protocol. For treatment plans in each cohort, a single fraction dose of 1.8 Gy to a total dose of 45 (n = 9) to 50.4 Gy (n = 16) was prescribed.

Additionally to treatment by IMPT or VMAT, all patients received a vaginal boost by high-dose-rate brachytherapy according to the Groupe Européen de Curiethérapie and the European Society for Radiotherapy and Oncology (GEC-ESTRO) guidelines.

The pelvic skeleton and its subsites iliac, lumbosacral, and lower pelvis were delineated retrospectively according to the guidelines by Mell et al.^4^ The external contour of all bones within the pelvis was contoured as a surrogate for the BM in order to ensure consistency as well as reproducibility. Contouring was performed on the original planning CT datasets and doses were analyzed with the original planning parameters.

For each plan, volume of the whole pelvic skeleton, extending from the most cranial border of the planning target volume (PTV) to the inferior border of the ischial tuberosities, and its subsites receiving doses of 5 Gy (V5Gy), 10 Gy (V10Gy), 20 Gy (V20Gy), 30 Gy (V30Gy), 40 Gy (V 40Gy) as well as mean dose (Dmean) were extracted. The lumbosacral region was defined as to start from the most cranial border of the PTV at the level of the aortic bifurcation correlating to L 4/5 and included the whole sacral bone. For the patients who received paraaortal irradiation, the most cranial level was chosen at the level of the aortic bifurcation. The lower pelvis region includes the acetabula as well as os pubes and os ischii and concludes with the inferior border of the ischial tuberosities. There was no overlap between the subsites.

Integral dose (ID) represented the area under the dose–volume histogram (DVH) curve at all dose levels and was defined as the sum of mean dose multiplied by the volume receiving the respective dose, as determined from dose data. For this analysis, the simplified formula ID = D_mean_ × volume was used.

Acute hematological toxicity occurring during radio(chemo)therapy and up to 2 weeks after completion of radiation therapy was evaluated retrospectively from the institutional database and clinical follow-up assessments. Leukocyte count, platelet, and hemoglobin nadirs were recorded. Hematotoxicity grade was defined by the greatest toxicity in any of these categories. Treatment-related toxicity was documented according to Common Terminology Criteria for Adverse Events (CTCAE) classification (v3).

Sacral insufficiency fractures were evaluated in the IMPT group by follow-up MRI scans per the protocol of the APROVE study. Due to MRI not being implemented by default during follow-up, evaluation of sacral insufficiency fractures was not possible in the photon cohort.

Statistical analysis was conducted using SPSSv28 (IBM Corp., Cohortonk, New York). Analysis was performed using Chi² test, Mann–Whitney U test, and receiver operating characteristics (ROC) curve.

All analyses were performed following institutional guidelines and the Declaration of Helsinki of 1975 in its most recent version. Patient confidentiality was maintained by anonymizing patient data to remove any identifying information. The reporting of this study conforms to STROBE (Strengthening the Reporting of Observational Studies in Epidemiology) guidelines.^24^

## Results

Detailed patient characteristics of the IMPT cohort were already described elsewhere.^10^ An overview of patient characteristics of both cohorts is listed in Table 1. On average patients were 61 years old at the start of radiotherapy in both cohorts, ranging from 39 to 82 years in the VMAT group and from 33 to 78 years in the IMPT group. Prescription dose was matched with 9 patients receiving a total dose of 45 Gy (RBE) in each cohort and 16 patients receiving 50.4 Gy (RBE). In both cohorts, one patient received paraaortal irradiation with a total dose of 50.4 Gy RBE. Most patients with uterine cervical cancer of each group were treated by radiochemotherapy with weekly cisplatin 40 mg/m². The pelvic bone volume was similar in both groups (*p *= 0.151) with a median volume of 1424 ml (range 916-1653 ml) in the IMPT cohort and 1318 ml (1046-1730 ml) in the VMAT cohort.

**Table 1.** Patients and treatment characteristics of both cohorts.

		VMAT	IMPT
Age	Mean	61	61
	Min	39	33
	Max	82	78
Uterine cervical cancer	8 (32%)	8 (32%)	
Endometrial cancer	17 (68%)	17 (68%)	
Prescription dose	45 Gy	9 (36%)	9 (36%)
	50.4 Gy	16 (64%)	16 (64%)
Planning target volume size	average	1406 ccm	1059 ccm
Previous chemotherapy		8 (32%)	8 (32%)
Concurrent chemotherapy		7 (28%)	7 (28%)
Treatment interruption due to hematotoxicity		2 (8%)	0 (0%)
Adjuvant chemotherapy		2 (8%)	5 (20%)
Hematotoxicity	Yes	20 (80%)	9 (36%)
	No	5 (20%)	16 (64%)

**Figure 1.**
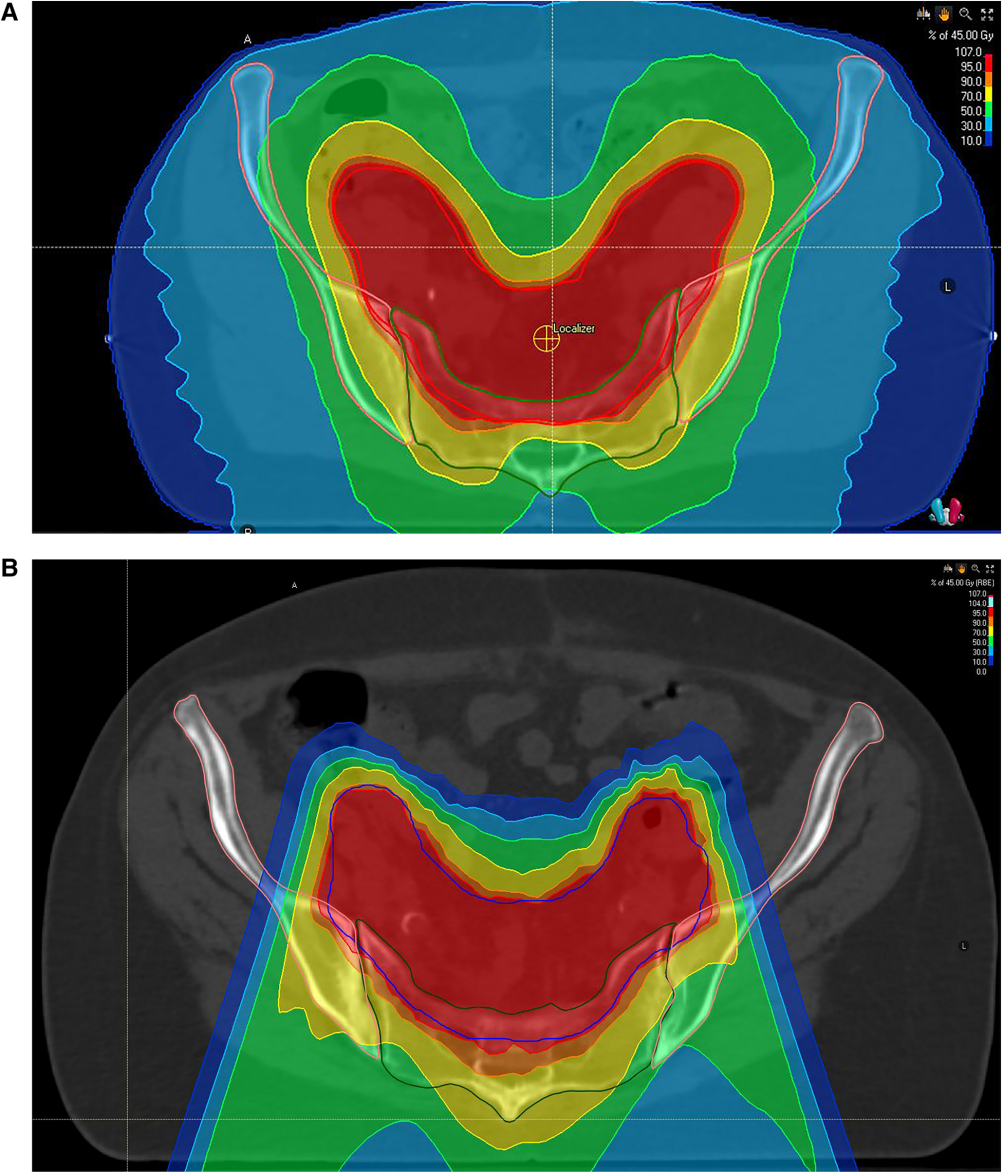
Representative treatment plans for both cohorts (A) VMAT, (B) IMPT. PTV is delineated in red, iliac contour is shown in pink, and sacral bone contour is presented in green. The isodoses are displayed in percentile of the prescription dose. 10%, 30%, 50%, 70%, 90%, 95%, and 107% isodoses are depicted.

Representative treatment plans with delineated bone contours are displayed in Figure 1 for both radiotherapy techniques.

Integral dose in the pelvic bone was significantly reduced in the IMPT cohort with an average integral dose of 23.4 Gy (range 9.2-53.3 Gy) compared to 34.3 Gy (range 24.5-45.3 Gy) in the VMAT group (*p *< 0.001). Detailed dosimetric information about the IMPT cohort is described elsewhere.^10^ In the IMPT group, the average V_5Gy_, V_10Gy_, and V_20Gy_ of the entire pelvic skeleton were reduced by 40%, 41%, and 28% compared to the VMAT group (*p* < 0.001). Especially for the subsites ilium and lower pelvis, the low dose exposure was significantly lower with a reduction of the average V_5Gy_, V_10Gy_, and V_20Gy_ by 39%, 42%, and 21% for the iliac bones and by 63%, 64%, and 56% for the lower pelvis. For the lumbosacral subsite, there was a significant reduction of V_5Gy_, V_10Gy_, and V_45Gy_ by 4%, 9%, and 38%, but there was a trend toward an increased V_30Gy_ by 10% (*p* = 0.109).

Dosimetric parameters and comparison between the modalities are displayed in Table 2.

**Table 2.** Mean values for the individual dose parameters of the VMAT compared to the IMPT cohort as well as the relative differences between the cohorts and Mann–Whitney U Test results (Table 2a: lumbosacral subsite and iliac subsite; Table 2b: pelvic skeleton and lower pelvic subsite).

	Lumbosacral				Ilium			
	VMAT (%)	IMPT (%)	Relative difference (%)	*p*	VMAT (%)	IMPT (%)	Relative difference (%)	*p*
V5Gy	96	92	−4	<0.001*	100	61	−39	<0.001*
V10Gy	98	89	−9	<0.001*	98	57	−42	<0.001*
V20Gy	84	83	−1	0.634	72	51	−29	<0.001*
V30Gy	61	67	+10	0.109	34	37	+9	0.174
V40Gy	38	33	−13	0.113	15	17	+13	0.251
V45Gy	24	14	−4	0.004*				
V50Gy	8	3	−9	0.002*				

	Pelvic skeleton				Lower pelvic			
	VMAT (%)	IMPT (%)	Relative difference (%)	*p*	VMAT (%)	IMPT (%)	Relative difference (%)	*p*
V5Gy	96	58	−40	<0.001*	90	33	−63	<0.001*
V10Gy	91	54	−41	<0.001*	81	29	−64	<0.002*
V20Gy	67	48	−28	<0.001*	52	23	−56	<0.003*
V30Gy	40	37	−8	0.066	30	17	−43	<0.004*
V40Gy	22	18	−18	0.015*	17	9	−47	<0.005*
Vol. cm³	1340	1382	−3	0.151				
Integral Dose in Gy(RBE)	34.3	23.4	−32	<0.001*				

Detailed toxicity analysis for the IMPT cohort has already been published.^25^ Hematological toxicity in both cohorts was analyzed and is displayed in Table 3.

**Table 3.** Acute haematological toxicity from the start and until 2 weeks after completion of radiation therapy.

	Grade 0		Grade 1		Grade 2		Grade 3	
	VMAT	IMPT	VMAT	IMPT	VMAT	IMPT	VMAT	IMPT
Anemia	11 (44%)	17 (72%)	12 (48%)	6 (24%)	2 (8%)	1 (4%)	0 (0%)	0 (0%)
Leukopenia	11 (44%)	19 (76%)	7 (28%)	3 (12%)	3 (12%)	3 (12%)	4 (16%)	0 (0%)
Thrombopenia	18 (72%)	25 (100%)	7 (28%)	0 (0%)	0 (0%)	0 (0%)	0 (0%)	0 (0%)

Data presented as number of patients, with percentages in parentheses.

In the VMAT cohort, anemia CTCAE grade 1 was preexistent in seven patients, deteriorating to a grade 2 anemia in two patients, staying at grade 1 for four patients, and normalizing in the course of radiotherapy for one patient. Eight further patients developed a grade 1 anemia during the course of treatment.

Seven patients developed leukopenia grade 1 during the course of treatment. Leukopenia grade 2 occurred in three cases, one of these patients had already had chemotherapy-associated leukopenia CTCAE grade 1 before the start of radiotherapy. In four cases leukopenia grade 3 was observed. 7 patients developed thrombopenia grade 1. All patients who developed a CTCAE grade 3 hematotoxicity received concurrent chemotherapy with weekly cisplatin 40 mg/m². Two of the patients in the VMAT cohort could only receive three cycles of chemotherapy due to hematotoxicity.

In the IMPT cohort, no patient experienced grade 3 hematotoxicity and only three cases of hematotoxicity grade 2 were observed. At the start of radiotherapy, anemia CTCAE grade 1 was pre-existent in six patients and one patient already had anemia grade 2. For five of these patients, anemia remained at grade 1. Two patients developed anemia grade 1 in the course of IMPT, both received chemotherapy. For two patients, hemoglobin levels normalized in the course of IMPT. The patient with preexisting grade 2 anemia received blood transfusion at the last day of therapy at a hemoglobin level of 8.4 g/dl. Another patient received blood transfusion at the first day of treatment at a hemoglobin level of 9.7 g/dl.

Leukopenia CTCAE grade 1 was pre-existent in four patients at the start of radiotherapy. In two of these patients, leukocyte count normalized during the course of radiotherapy, the other two patients developed grade 2 leukopenia during or after completion of radiotherapy. In one further patient, grade 2 leukopenia was observed shortly after completion of treatment. All of the patients experiencing grade 2 leukopenia had simultaneous or preceding chemotherapy. Two patients developed grade 1 leukopenia in the course of treatment.

All of the seven patients who received concurrent chemotherapy received the full dosage cisplatin 40 mg/m²weekly.

Myelosuppression during and until 2 weeks after completion of radiotherapy was significantly more common in the VMAT cohort affecting 80% of the patients compared to 36% in the IMPT cohort (*p *= 0.004, Chi² test).

**Figure 2.**
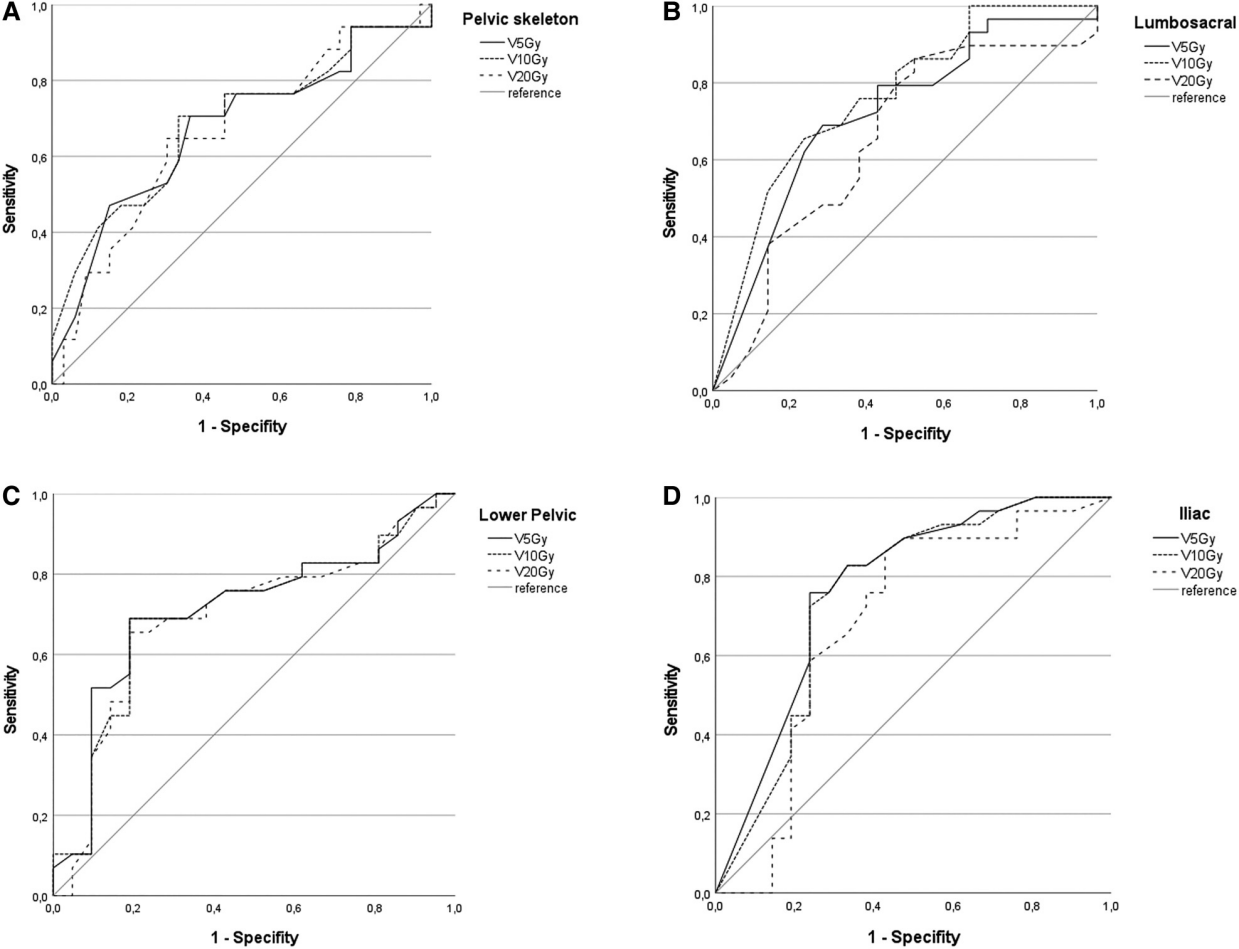
ROC Curve for prediction of occurrence of hematotoxicity of any CTCAE grade in relation to V5Gy, V10Gy, and V20Gy to the pelvic skeleton (A), to the lumbosacral subsite (B), to the lower pelvic subsite (C), and to the iliac subsite (D).

ROC and area under the curve (AUC) analysis was performed to identify dosimetric predictors for the occurrence of myelosuppression. For the pelvic skeleton, AUC was 0.736 (V5Gy, *p *= 0.005), 0.735 (V10Gy, *p *= 0.005) and 0.707 (V20Gy, *p *= 0.013) regarding any hematotoxicity. For the lower pelvic subsite, AUC was 0.712 (V5Gy, *p *= 0.011), 0.702 (V10Gy, *p *= 0.,016), and 0.694 (V20Gy, *p *= 0.020) and for the iliac subsite, AUC was 0.771 (V5Gy, *p *= 0.001), 0.754 (V10Gy, *p *= 0.002), and 0.685 (V20Gy, *p *= 0.027), respectively. For the lumbosacral subsite, only V5Gy (AUC 0.718, *p *= 0.009) and V10Gy (AUC 0.762, *p *= 0.002) had a predictive value for the occurrence of hematotoxicity, while for V20Gy AUC was 0.651 (*p *= 0.082). The corresponding curves are depicted in Figure 2.

Since MRI-based follow-up was only available for 3 of the 25 patients treated with VMAT, analysis of occurrence of sacral insufficiency fractures was not reasonable for this cohort. All patients treated by IMPT received follow-up by MRI per protocol of the APROVE trial. Out of 25 patients in the IMPT cohort, 8 (32%) patients experienced sacral insufficiency fractures after completion of radiotherapy. Median time to development of SIF was 8 months (range 1−16 months). Diagnosis was based on MRI as part of the regular follow-up. Five patients were not symptomatic. Three patients had corresponding pain symptoms. One of these patients had already suffered from osteoporosis before and showed additional edema corresponding to insufficiency fractures in the pubic bones and needed analgetic medication on a regular basis. Two of the patients who showed SIF had not been treated with chemotherapy. The patients who developed SIF were on average slightly older than the patient without SIF with an average age of 63 years (vs 58 years) at the start of radiotherapy and showed a BMI ranging from 20 to 35 compared with 18 to 42. There were no significant correlations between age (*p *= 0.511), BMI (*p *= 0.598), or application of chemotherapy (*p *= 0.68) on occurrence of SIF.

**Figure 3.**
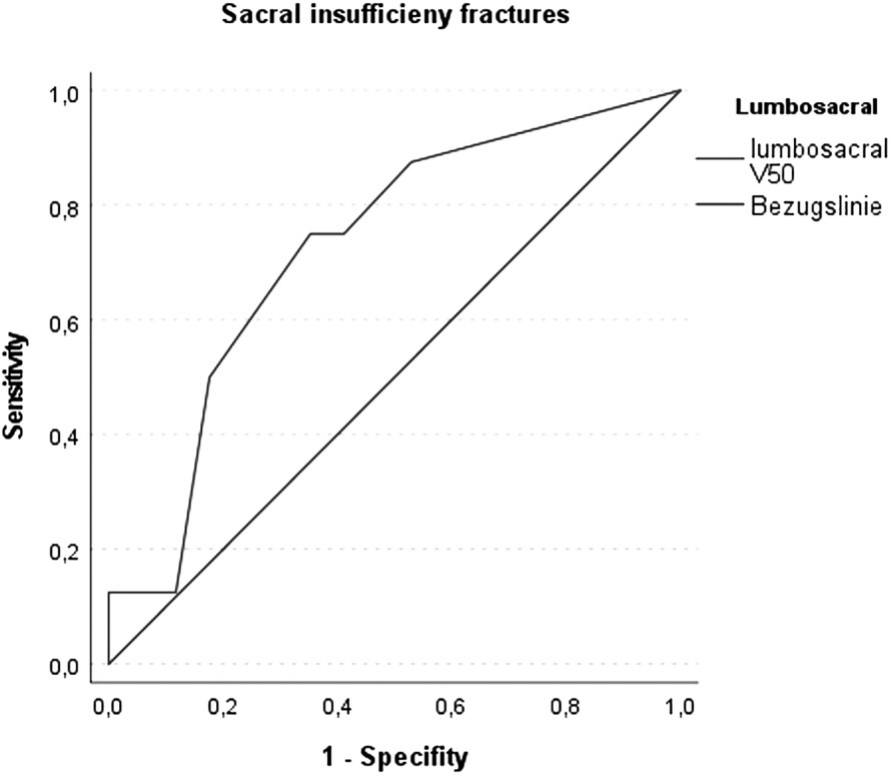
ROC Curve of V50Gy of the lumbosacral subsite as predictor for the occurrence of sacral insufficiency fractures. AUC is 0.724 (*p* = 0.076).

The V_5Gy_, V_10Gy_, V_20Gy_, V_30Gy_, or V_40Gy_ to the lumbosacral subsite or the sacral bone did not significantly differ between patients who suffered SIF and those who did not show any skeletal changes. SIF was noticeably more common in patients who had received a total dose of 50.4 Gy RBE: 6 out of the 16 patients (38%) compared to 2 out of 9 patients (22%) who had received a total dose of 45 Gy RBE. However, Mann–Whitney U testing of V50Gy (*p *= 0.075) and maximal dose (*p *= 0.075) to the lumbosacral subsite for correlation with occurrence of sacral insufficiency fractures only showed a trend for SIF with increased doses to the lumbosacral subsite. In the ROC analysis, AUC for V50 was 0.724 (*p *= 0.076), as depicted in Figure 3. There was no significant correlation of the mean dose (*p *= 0.288) or V45Gy (*p *= 0.588) with the occurrence of SIF.

## Discussion

Irradiation of the bones leads to apoptosis of BM and peripheral blood stem cells. The subsequent marrow stromal damage results in characteristic pathologic and radiographic bone changes.^26,27^ Marrow stem cells are sensitive to even modest doses of radiation, which is the primary reason of hematological toxicity of radiotherapy.^2,16,28^ Hematological toxicities often lead to missed chemotherapy cycles and treatment interruptions which may compromise a successful treatment and thus oncological outcome.^12,29^ Reducing lower dose (0-30 Gy) volumes with advanced radiotherapy techniques allows for balancing acute hematological toxicity, whereas reduction of high dose (>35 Gy) volume is essential for improved long-term BM recovery.^28^

Hematotoxicity is known to occur following postoperative radio(chemo)therapy for cervical and endometrial cancer patients using standard-of-care IMRT. For example, an analysis of the patient cohort in the PACER trial receiving postoperative bowel sparing IMRT in combination with concurrent weekly admission of cisplatin observed grade ≥2 leukopenia, neutropenia, anemia, and thrombocytopenia in 26%, 40%, 26.5%, and 1.4% of the patients, respectively.^30^ The RTOG 0418 trial, a phase 2 study of postoperative radio(chemo)therapy for cervical and endometrial cancer patients using IMRT compared to conventional 4-field irradiation, showed grades 1-5 hematotoxicity of 23%, 33%, 25%, 0%, and 0%, respectively, for patients receiving concurrent cisplatin and pelvic IMRT.^31^

In our previous publication, we demonstrated excellent treatment tolerability of IMPT in gynecological malignancies regarding gastrointestinal and urogenital toxicities.^22^ In this study, we specifically evaluated potential advantages of IMPT regarding pelvic skeleton dosimetry and thus hematotoxicity compared to state-of-the-art IMRT applying VMAT. Although there is an increasing understanding that RBE of protons actually varies along the beam path, a fixed RBE value of 1.1 was used for proton radiotherapy planning, since there is still uncertainty of the possible impact on current clinical practice.^32^In the literature, different dose parameters correlating with hematotoxicity by applying postoperative IMRT for gynecological malignancies have been described. Rose et al evaluated associations between hematologic nadirs during chemoradiotherapy in cervical cancer patients and the volume of BM receiving ≥10 Gy and 20 Gy (V10 and V20) using a previously developed linear regression model. They hypothesized that keeping V10 < 95% and V20 < 76% may reduce hematotoxicity.^12^ Lewis et al reported that V40 > 35% of the whole pelvis bony structures and V40 > 20% of the lower pelvis bony structures correlates with ≥grade 2 leukopenia and neutropenia, but none of the DVH parameters could be validated on multivariate analysis.^30^ Klopp et al observed that the median BM dose of >34.2 Gy and V40 > 37% correlated significantly with higher rates of grade ≥2 hematotoxicity.^31^

Proton therapy offers superior dose distribution characteristics for BM sparring compared to conventional photon-based techniques. There are few planning studies evaluating doses to the pelvic skeleton by using proton beam therapy. For example, Lin et al treated 11 patients with posthysterectomy gynecologic cancer with proton beam therapy using a 2-field posterior oblique beam with pencil beam scanning proton beam radiation therapy (PBS). For each patient, a dosimetric comparison between the PBS and an IMRT plan was conducted. They could show that the V10 or V20 of the pelvic BM were significantly higher with IMRT than with the posterior PBS technique but IMRT was significantly better at sparing the total pelvic BM in the high-dose region (>35 Gy). They further examined the dose to the pelvic substructures and found the ilium and lower pelvis V10 and V20 to be significantly lower with PBS than with IMRT, whereas the V12-V50 of the lumbosacral spine was significantly lower with IMRT. Of the 11 patients, 1 developed grade 3 leukopenia and 3 grade 2 leukopenia.^33^ A dosimetric comparison of different IMRT and IMPT plans of 33 patients with cervical cancer by Qin et al showed that compared to IMRT, bone-marrow-sparring IMPT reduced the V5, V10, V20, V30, V40 Gy, and Dmean of the high-active BM by 10.2%, 36.8%, 58.8%, 67.4%, 64.9%, and 44.5%, respectively.^34^

Even in extended field treatment of gynecological malignancies, IMPT resulted in lower volumes of exposed pelvic marrow.^35,36^ In the study by Xu et al, PBS resulted in 22% lower median pelvic BM volume irradiated to 10 Gy (RBE) and 14% lower median volume irradiated to 20 Gy (RBE).^36^

In our study, we also found that IMPT significantly reduced the low-dose exposure (V5-V20) to the whole pelvic skeleton, in the lower pelvic structures even V30 and V40 could be significantly reduced. For the lumbosacral subsite a reduced V5, V10, V45, and V50 could be observed, though the average V30Gy was elevated (non-significantly). These data are in accordance with those from Lin et al. The reason for the similar or even higher high dose volumes with IMPT is probably the use of posterior oblique beams and the need to pass through the sacrum, which has also been described by Lin et al.^33^

We demonstrated a significant reduction in hematotoxicity by the use of IMPT in comparison to treatment by VMAT in a matched-pair analysis (*p *= 0.004). In general, rates of hematotoxicity were very low in the IMPT group, no hematotoxicity exceeding grade 2 was observed. In the IMRT cohort, the incidence of hematotoxicity was significantly higher, with 16% of patients developing grade 3 leukopenia. Consequently, concomitant chemotherapy had to be stopped after three cycles in two of the eight patients who were treated with simultaneous cisplatin 40 mg/m² weekly. This suggests, that the reduction of the low dose exposure to the pelvic skeleton with IMPT might improve compliance with chemotherapy treatment protocols by reducing hematotoxicity.

One challenge in comparing literature is the inconsistent definition or delineation of pelvic BM. In our study, we took the pelvic skeleton as a surrogate for the pelvic BM according to the data by Mell et al. The external contour of all bones within the pelvis was delineated on the planning CT scan. The advantage of using the external contours of the bones is to ensure reproducibility and to minimize dependence of the contours on CT windowing and leveling.^3^ In the RTOG 0418 trial, pelvic BM was defined within the treatment field by using a CT density-based autocontouring algorithm.^31^ However, we found that degenerative bone changes increased the bone volumes and might misrepresent the assumed BM proportions. For further investigations, different methods of BM contouring should be considered.

Another refined way to define functional BM is to use functional MR imaging or positron emission tomography (PET) scans to delineate active BM. For instance, Rose et al employed a 18F-FDG-PET-based definition to identify active BM. According to their findings, the predicted threshold for avoiding grade 3 leukopenia was a mean dose of less than 26.8 Gy to the active BM. They also observed significant associations between an increased dose to the active BM and a decreased white blood cells nadir.^5^ Qin et al used functional MR imaging for definition of functional BM. The pelvic BM was contoured on MRI sequences that were reconstructed to obtain the fat fraction of tissue and were categorized into high-active BM and low-active BM based on the fat content of the pelvic BM.^34^

There is a huge variation of reported rates of pelvic insufficiency fractures after radiotherapy in the literature.^37^ Only about half of the patients with changes to the bone structure on MRI develop symptoms and most cases can be treated conservatively.^11,37^ Various authors have tried to understand the predictive factors for sacral insufficiency fractures.^11,27,37–40^ According to Mir et al age and V40Gy are predictors for insufficiency fractures. In their cohort treated by photon beam radiotherapy, 37.4% developed sacral insufficiency fractures evaluated by follow-up MRI; 93.0% were detected within 12 months of external beam radiotherapy. In our IMPT cohort, 32% developed SIF within 24 months after proton beam therapy. The use of posterior oblique beams in our study, with similar high dose volume due to beam entry through the sacrum, might be the reason for similar rates of sacral insufficiency fractures in both cohorts. We found that there was a corresponding trend for a correlation of SIF with a prescribed dose of more than 50 Gy RBE (*p *= 0.075). Oh et al analyzed 557 patients who received whole-pelvic radiotherapy and found significant correlation of SIF with prescribed dose of more than 50.4 Gy (*p* = 0.04).^39^ Another risk factor for the occurrence of SIF regardless of pelvic irradition is osteoporosis.^41^ There was no data on bone density available for analysis for the cohort of this study.

Pelvic BM is usually not taken as a planning constraint in routine practice as it risks increase in radiation dose to other pelvic organs. In our case, even without taking BM into consideration prospectively, we achieved lower doses when applying proton instead of photon radiotherapy. This dosimetric advantage translates into clinical reduction of acute hematological toxicities, as demonstrated in our study. As the risk of sacral insufficiency fractures seems to be dependent on the high dose to the sacrum, no advantage of IMPT could be discovered compared to IMRT.

Nonetheless, conducting further studies with a prospective setting would be desirable to strengthen and validate these findings. Results from the ongoing prospective phase-II PROTECT trial evaluating the benefit of adaptive proton radiotherapy for cervical cancer, are anticipated to provide additional insights into the BM sparing capacity of both radiotherapy techniques.^42^

Limitations to the current study were mainly caused by the retrospective nature of the analysis and the relatively small sample size which was due to a limited number of patients with gynecological malignancies treated by IMPT at the Heidelberg Ion Therapy Center. Furthermore, the patient population was somewhat inconsistent in terms of prescription dose, application of chemotherapy, and disease histologies as well as stage. To account for these limitations the control cases of the case–control study were selected in such a way that the greatest possible agreement could be achieved regarding these factors. The analyzed cohorts were well balanced in this regard. It is important to mention that the treatment volumes in the IMPT cohort were on average slightly smaller compared to the VMAT cohort, which could potentially influence the observed differences in dose exposure to the organs at risk. However, considering the substantial differences in low-dose exposure to the lower pelvis and iliac subsites, the small differences in PTV sizes can likely be disregarded. Additional limitations of our results include the retrospective nature of the hematotoxicity analysis, as the timing of blood counts were not consistent. Furthermore, the lack of MRI-based follow-up in the VMAT cohort hinders a comprehensive analysis of sacral insufficiency fractures. These limitations should be taken into consideration when interpreting the findings of this study.

## Conclusion

The use of intensity-modulated proton beam therapy can considerably reduce dose exposure to the pelvic skeleton, especially to the lower pelvis and ilium compared to conventional photon radiotherapy. By applying IMPT, hematological toxicity of postoperative radio(chemo)therapy for gynecological malignancies may be significantly reduced. This may be advantageous in terms of concurrent and adjuvant chemotherapy. But correspondingly, no reduction in the high dose volume to the sacral region was detected, possibly due to entry dose of posterior oblique fields in IMPT. This is probably the reason why sacral insufficiency fractures occured at similar rates to reported data on patients treated by IMRT. More prospective studies are required to validate these findings.

## Abbreviations

AE: Adverse event
AUC: Area under the curve
CT: Computer tomography
CTCAE: Common terminology Criteria for Adverse Events
IMPT: Intensity-modulated proton therapy
MRI: Magnetic resonance imaging
PBS: Pencil beam scanning
PET: Positron emission tomography
PTV: Planning target volume
RBE: Relative biological effectiveness
ROC: Receiver operating characteristic
STOBE: Strengthening the Reporting of Observational Studies in Epidemiology
VMAT: Volumetric target volume
